# The role of breast tomosynthesis in a predominantly dense breast population at a tertiary breast centre: breast density assessment and diagnostic performance in comparison with MRI

**DOI:** 10.1007/s00330-017-5297-7

**Published:** 2018-02-19

**Authors:** Daniel Förnvik, Masako Kataoka, Mami Iima, Akane Ohashi, Shotaro Kanao, Masakazu Toi, Kaori Togashi

**Affiliations:** 1 Medical Radiation Physics, Department of Translational Medicine, Faculty of Medicine, Lund University, Skåne University Hospital, 205 02 Malmö, Sweden; 2 Department of Diagnostic Imaging and Nuclear Medicine, Graduate School of Medicine, Kyoto University, 54 Shogoin-Kawaharacho Sakyo-ku, Kyoto, 606-8507 Japan; 3 Department of Breast Surgery, Graduate School of Medicine, Kyoto University, 54 Shogoin-Kawaharacho Sakyo-ku, Kyoto, 606-8507 Japan

**Keywords:** Breast cancer, Breast density, Digital breast tomosynthesis, Magnetic resonance imaging, Diagnostic techniques and procedures

## Abstract

**Objectives:**

To compare breast density measured on digital breast tomosynthesis (DBT) (BI-RADS-based breast composition and fully-automatic estimation) and magnetic resonance imaging (MRI) (BI-RADS amount of fibroglandular tissue), and to evaluate the diagnostic performance in terms of sensitivity and specificity of DBT and MRI in a predominantly dense breast population.

**Methods:**

Between 2015 and 2016, 152 women with 103 breast malignancies, who underwent 3-T breast MRI and DBT within 2 months’ time, were enrolled in this study. Breast composition/fibroglandular tissue and findings on DBT (two readers) and MRI were reported using BI-RADS 5th edition. Digital mammography images were analysed for breast percent density (PD) using the Libra software tool.

**Results:**

A majority of women had dense breasts as categorised by breast composition c (heterogeneously dense) (68%) and d (extremely dense) (15%). The mean PD was 44% (range, 18-89%) and the correlation between breast composition and PD was *r* = 0.6. The diagnostic performance of MRI was significantly higher compared to DBT for one reader as described by the area under the receiver operating characteristic (ROC) curve (*p* = 0.004) and of borderline significance for the other reader (*p* = 0.052).

**Conclusions:**

MRI had higher diagnostic performance than DBT in a dense breast population in the tertiary setting.

**Key Points:**

*• MRI had higher diagnostic performance than DBT in a dense breast population*

*• Diagnostic performance of DBT was comparable to MRI in women with fatty breasts*

*• MRI was superior to DBT in preoperative breast cancer size assessment*

## Introduction

Digital breast tomosynthesis (DBT) has gained increasing interest in the clinic for routine everyday use, with several early clinical studies showing superior accuracy compared to that of mammography [1–3]. Large multicentre studies have shown general benefits of DBT over digital mammography (DM), but the role of tomosynthesis in women with dense breasts has not yet been fully established [4–6]. Prospective trials of screening a population from Europe show a statistically significant increase in cancer detection rate with DBT (with or without combined DM) compared with two-view DM independent of vendor system [7–9]. Skaane et al. [7] stated: “Notably, the additional cancers detected with mammography plus tomosynthesis were distributed across all breast densities, including fatty breasts”. Lång et al. [9] reported that the additional cancers were detected both in women with dense and fatty breasts, drawing the conclusion that in mammography even a moderate amount of breast tissue can conceal a small lesion.

Breast cancer risk increases with increasing breast density [10, 11]. The relationship holds true also for Japanese women [12, 13], who are known to have dense breasts [14] and highest age-specific breast cancer incidence at age 45–49 [15]. Breast density has until recently been classified qualitatively by use of the four categories in the ACR Breast Imaging Reporting and Data System (BI-RADS) coding system: (1) almost entirely fat (<25% fibroglandular tissue), (2) scattered fibroglandular densities (25-50% fibroglandular tissue), (3) heterogeneously dense (51-75% fibroglandular tissue) and (4) extremely dense (>75% fibroglandular tissue) [16]. Recent revision of BI-RADS concerns changes to category 3: the breasts are heterogeneously dense, which may obscure small masses, and discourage the use of a cut-off based on percent density [17]. In order to more objectively assess breast density and to reduce interobserver and intraobserver variability, quantitative measurements have been developed [18, 19]. Both qualitative and quantitative methods of measuring breast density have shown an association between high breast density and breast cancer risk [11, 20].

More than half of the USA states have begun legislating mammographic breast density reporting to women, requiring that women be notified of breast density with their mammography results [21]. As a result, the U.S. Preventive Service Task Force conducted a review of supplemental screening for breast cancer in women with dense breasts and concluded that density ratings may be recategorised on serial screening mammography and, with limited evidence, that additional imaging with DBT reduces recall rates in women with dense breasts; however, it was not clear the results implied fewer overall breast biopsies [22].

Dynamic contrast-enhanced breast (DCE) MRI has excellent sensitivity independent of breast density but still moderate specificity [22, 23]. By adding diffusion-weighted imaging (DWI) sequences, there is optimism to increase the specificity [24]. Recommended indications for DCE MRI on defined subgroups include: screening of high-risk women due to cancer susceptibility genes or greater than 20% lifetime risk of developing breast cancer, assessment of occult primary breast cancer, preoperative staging and evaluation of neoadjuvant therapy [25, 26]. MRI is expensive and time-consuming, hence the use of MRI in the clinic varies mainly due to accessibility.

The purpose of this study was to compare breast density measured on DBT and MRI, and to evaluate the diagnostic performance in terms of sensitivity and specificity of DBT and MRI in a predominantly dense breast population in the tertiary setting.

## Materials and methods

### Patient population and lesions

Approval was obtained from the Institutional Review Board of our institution with a waiver of informed consent due to the retrospective nature of the study. Between March 2015 and March 2016, a total of 494 patients underwent 548 MRI examinations and 2,164 patients underwent 2,292 DBT examinations at the Department of Radiology, Kyoto University Hospital, Japan. The inclusion criteria included matched MRI/DBT patient examinations within 2 months’ time (*n* = 249) and BI-RADS diagnostic category 3, 4 or 5 on MRI, resulting in 162 patients. Of these, 10 were excluded from analysis because of previous breast surgery (*n* = 5), incomplete MRI examination (*n* = 2), insufficient follow-up (*n* = 2) or Paget’s disease (*n* = 1). In total, 152 female patients (mean age, 57.1 ± 13.8 years; range, 28-83 years), of which 56 were healthy and 96 presented with 103 breast malignancies including 4 bilateral and 3 multicentric, were enrolled in this study. All breast cancer diagnoses were verified by surgery and/or biopsy. Patients were considered healthy after negative surgery and/or biopsy or at least 1-year negative imaging follow-up on digital mammography and ultrasound (US) and/or MRI. The MRI sample represented 31% (152/494) and the DBT sample 7% (152/2,164) of the total number of women being examined during the actual period of time. MRI was performed on average 8.7 ± 10.6 days (range, -46 to 50 days) after DBT.

All women referred for clinical assessment during the time period of the study had DBT as part of their examination, unless contraindicated or DBT was recently acquired at another hospital. Indications for MRI in this study population were mostly problem solving (*n* = 130; 85.5%) with the following details: suspicious finding by DM (*n* = 29; 19.1%), suspicious finding by US (*n* = 59; 38.8%), suspicious finding by DM and US (*n* = 31; 20.4%), suspicious finding by computed tomography (*n* = 11; 7.2%). Other indications included high-risk women (*n* = 2; 1.3%) and preoperative staging (*n* = 20; 13.2%).

### Imaging acquisition

All patients included underwent bilateral two views (craniocaudal and mediolateral oblique) using a full-field DM unit with tomosynthesis capability (Selenia Dimensions; Hologic, Bedford, MA, USA) operating in combo mode: DM and DBT images were obtained within a single breast compression for each projection. W/Rh and W/Al were used as anode/filter combinations for DM and DBT, respectively, automatic exposure control was employed and 15 low-dose images were acquired during the X-ray movement over an angular range of 15°. These low-dose images were reconstructed into millimetre-thin slices of the breast using filtered back-projection. The mean compressed breast thickness was 34.4 ± 12.6 mm (range, 8.5-73.5 mm).

The MRI examinations were performed with a 3.0-T scanner (MAGNETOM Trio, A Tim System; Siemens, Erlangen, Germany) with 16/4 channel breast coil. The parameters were as follows: T2-weighted images (whole breast; axial orientation; 2D-turbo spin echo with fat suppression; repetition time/echo time (TR/TE), 5,500/70 ms; field-of-view (FOV), 330 × 330 mm; matrix, 448 × 336; thickness, 3.0 mm), T1-weighted images (whole breast; axial orientation; 3D volumetric interpolated breath-hold examination (3D-VIBE); TR/TE, 4.95/2.46 ms; FOV, 330 × 330 mm; matrix, 480 × 398; thickness, 1.5 mm), T1-weighted DCE images scanning at pre-contrast state and at 0–1, 1–2 and 5–6 min after gadolinium injection (whole breast; axial orientation; 3D-VIBE with fat suppression; TR/TE, 3.95/1.43 ms; flip angle (FA), 15°; FOV, 330 × 330 mm; matrix, 384 × 346; thickness, 1.0 mm), contrast-enhanced T1-weighted images in high spatial resolution at 2–4.5 min after gadolinium injection (whole breast; coronal orientation; 3D-VIBE with fat suppression; TR/TE, 4.61/1.81 ms; FA, 15°; FOV, 330 × 330 mm; matrix, 512 × 461; thickness, 0.8 mm). The MR examination was performed during first half of the menstrual cycle in premenopausal women and without scheduling limitations in postmenopausal women.

### Image interpretation and analysis

Two breast radiologists (M.I. and M.K. with 10 and 18 years of experience in mammography, respectively) reviewed and assessed the DBT images according to BI-RADS 5th edition on a dedicated workstation unaware of any clinical information or the histopathology diagnosis [17]. Breast composition (based on mammography projections) and findings on DBT were characterised and recorded. BI-RADS categories 1-3 were considered benign, and BI-RADS categories 4 and 5 were considered malignant. The MR images were assessed and approved at the time of clinical interpretation by one of two specialised breast radiologists (M.K. and S.K.) in accordance with BI-RADS. Fibroglandular tissue (FGT), background parenchymal enhancement (BPE), findings and enhancement characteristics, including kinetic curve assessment, were characterised and recorded. The software package LIBRA, a fully-automatic breast density estimation software solution, was used in this study to estimate the percentage breast density (PD) [27, 28]. The average PD of the four DM images was calculated and in cases containing larger (>30 mm) invasive tumours, only the average of the contralateral values was used (the size cut-off was chosen not to exceed the natural dense breast tissue variation between the two breasts, data not shown).

A preoperative tumour size assessment was performed by retrospectively measuring the largest lesion diameter to the nearest millimetre at the central core on both DBT and MR images to establish which imaging modality most accurately corresponds with the size of the pathology report [29, 30]. Tumours were classified as not measurable in cases of architectural distortion without definable borders or in cases where the tumour was partially obscured by dense tissue on DBT and in cases of multiple or diffuse enhancement patterns (mostly classified as non-mass enhancement) within the breast on MRI. Only invasive cancers were included in the analysis.

### Statistical analysis

Inter-rater reliability was measured using percent agreement and weighted kappa statistics. Pearson’s *r* and Spearman’s ρ were used to test for correlations for normally and skewed distributed data, respectively. One-way analysis of variance with Bonferroni correction was used to compare PD means between groups. Diagnostic performance was assessed with receiver operating characteristics (ROC) curve analysis [31]. Area under the curve (AUC) was calculated from both parametric and trapezoidal curve fitting. Fisher’s exact test and logistic regression analysis were performed to determine which variables are associated with false negative (FN) outcomes. Size agreement between imaging modality and pathology measurements were analysed following the approach of Bland and Altman. All analyses were performed using the SPSS software (version 24; IBM Corp., Armonk, NY, USA) and *p* values <0.05 were considered statistically significant.

## Results

Out of all 103 breast cancers, 64 were invasive carcinoma of no special type (NST), 26 ductal carcinoma in situ (DCIS), 6 invasive lobular carcinoma (ILC), 4 mucinous carcinoma, 1 micropapillary carcinoma, 1 tubular carcinoma and 1 apocrine carcinoma.

The average percentage distribution of breast composition (based on DBT) was: 2.3% (*n* = 3.5) category a, 15.5% (*n* = 23.5) category b, 67.8% (*n* = 103) category c, 14.5% (*n* = 22) category d. There was substantial inter-rater agreement between the two readers (R1 and R2) with percent agreement of 85% (129/152) and weighted κ = 0.74 [95% confidence interval (CI), 0.64-0.84].

The percentage distribution of amount of FGT (based on MRI) was: 5.9% (*n* = 9) a. Almost entirely fat, 30.3% (*n* = 46) b. Scattered fibroglandular tissue, 53.3% (*n* = 81) c. Heterogeneous fibroglandular tissue, 10.5% (*n* = 16) d. Extreme fibroglandular tissue.

The percentage distribution of background parenchymal enhancement (BPE) level (based on MRI) was: 22.1% (*n* = 31) minimal, 32.1% (*n* = 45) mild, 26.4% (*n* = 37) moderate, 19.3% (*n* = 27) marked.

**Fig. 1:**
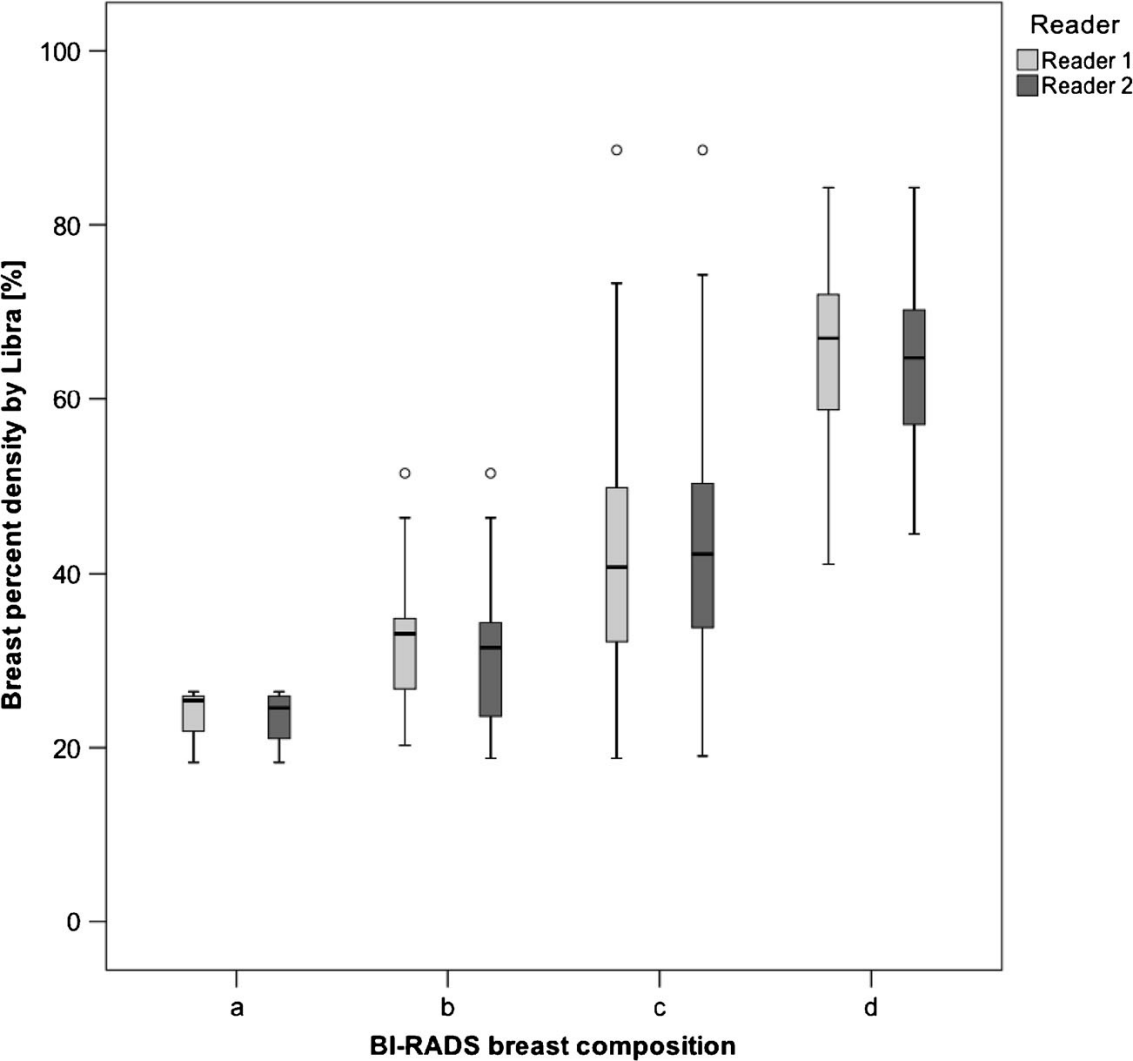
BI-RADS breast composition categories with corresponding breast percent density as estimated by Libra

The mean PD was 43.5% (range, 18.3-88.6%). The average mean PD for the different breast compositions was: a = 23.4% (range, 18.3-26.4%), b = 31.7% (range, 18.8-51.5%), c = 42.4% (range, 18.8-88.6%) and d = 64.8% (range, 41.1-84.3%) (Fig. 1). All category means were significantly different (*p* < 0.009), except for a with b (*p* = 1.00).

The correlations between breast composition, FGT, BPE and PD ranged from weak to strong (Table 1).

**Table 1:** Bivariate correlations

	FGT	BPE	PD
Breast composition Reader 1	0.53 (0.40-0.64)	0.28 (0.11-0.45)	0.57 (0.46-0.67)
Breast composition Reader 2	0.54 (0.41-0.65)	0.28 (0.09-0.44)	0.63 (0.53-0.72)
FGT		0.37 (0.22-0.53)	0.51 (0.38-0.63)
BPE			0.37 (0.22-0.51)

*Numbers in parenthesis* are 95% CIs

*FGT* fibroglandular tissue, *BPE* background parenchymal enhancement, *PD* percent density

**Fig. 2:**
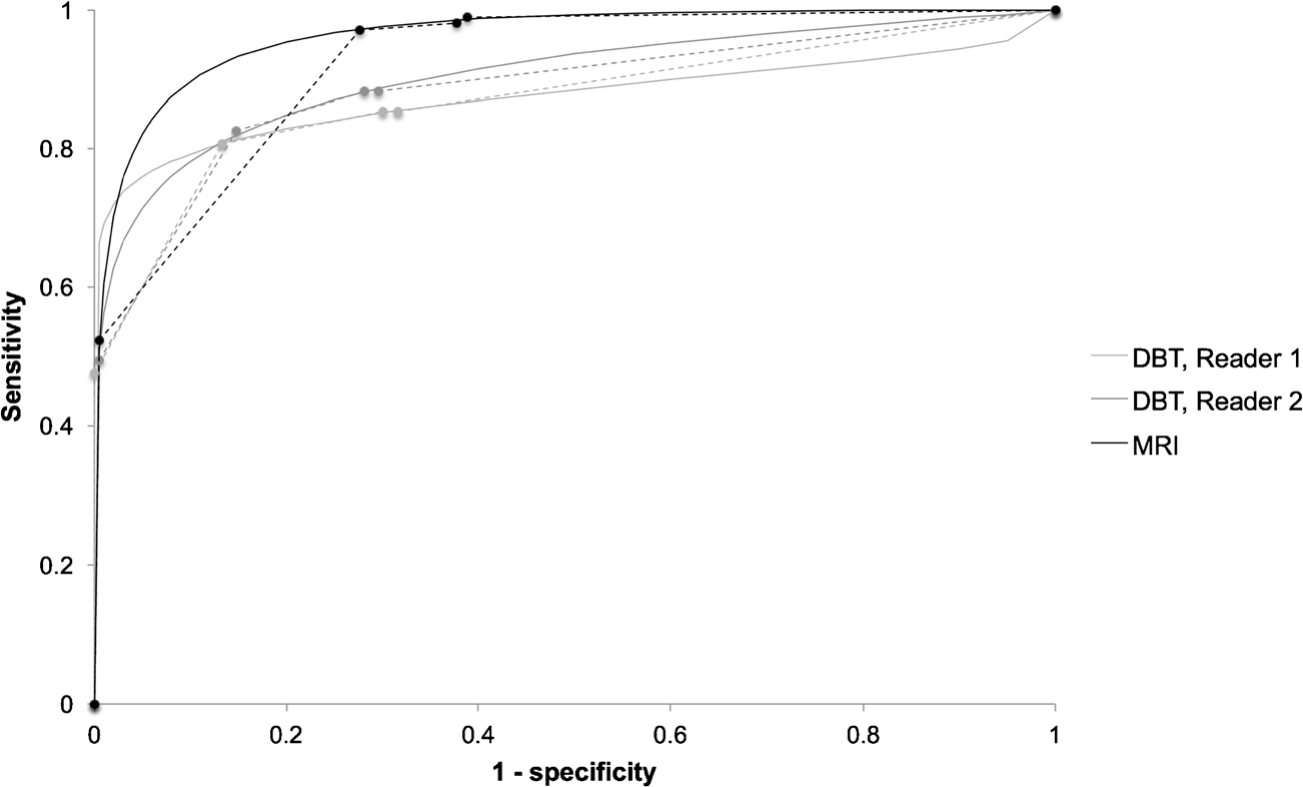
ROC curves for DBT and MRI. *Solid lines* parametric model, *dashed lines* trapezoidal model

On DBT, R1 had a sensitivity of 80.6% (FN = 20) and positive predictive value (PPV) of 76.1% (FP = 26) and R2 had a sensitivity of 82.5% (FN = 18) and PPV of 74.6% (FP = 29). The AUC of the parametric ROC curve was 0.875 (95% CI, 0.801-0.927) and 0.906 (95% CI, 0.852-0.944), and the AUC of the trapezoidal ROC was 0.872 (95% CI, 0.823-0.920) and 0.886 (95% CI, 0.841-0.930) for R1 and R2, respectively (Fig. 2). MRI had a sensitivity of 97.1% (FN = 3) and PPV of 62.5% (FP = 60). The AUC of the parametric ROC curve was 0.964 (95% CI, 0.931-0.983) and the AUC of the trapezoidal ROC was 0.922 (95% CI, 0.893-0.952). The diagnostic performance of MRI was significantly higher compared to DBT for R1 using the parametric model (*p* = 0.004) and borderline higher compared to DBT for R2 (*p* = 0.052). R2 rated one false positive (FP) as BI-RADS 5 on DBT and one case of FP was rated as BI-RADS 5 on MRI. Twenty-five percent (*n* = 5) and 33% (*n* = 6) of the FN on DBT were rated as BI-RADS 3 by R1 and R2, respectively, and 33% (*n* = 1) of the FN on MRI was rated as BI-RADS 3.

By substituting MRI with DBT for women with mammography breast composition category a or b, the MRI FP could be reduced on average by 6% (R1, *n* = 4, R2, *n* = 3) at the cost of a 1% average reduced sensitivity (R1, *n* = 0; R2, *n* = 2). By applying PD threshold (<23.5%), a FP decrease of 3% (R1, *n* = 2; R2, *n* = 2) can be achieved at no sensitivity loss.

### Patient characteristics

In general, FP were highly correlated on the two systems, 77% (20/26, R1) and 76% (22/29, R2).

No patient characteristics, including PD, were associated with FN on DBT (Table 2). When dichotomised as fatty (category a + b) or dense (category c + d), breast composition suggested an association with FN on DBT, although not reaching statistical significance, for R1 (*p* = 0.080). There was no association with FN on DBT for R2 (*p* = 0.366). No FN on DBT was found in category a or b for R1 and two FNs in category b (rated as c by R1) for R2. The lowest PD encountered for an FN was 23.5% for both readers.

**Table 2:** Univariate associations of patient and lesion characteristics with false negatives on DBT

Patient characteristics	True positive		False negative		Significance^a^	
	R1	R2	R1	R2	R1	R2
Age			OR = 1.02 95% CI = 0.98-1.05	OR = 1.01 95% CI = 0.98-1.05	*p* = 0.334	*p* = 0.503
Breast composition					*p* = 0.155	*p* = 0.649
a (fatty)	3 (2)	4 (3)	0 (0)	0 (0)		
b	19 (14)	26 (19)	0 (0)	2 (11)		
c	93 (71)	86 (64)	15 (75)	12 (67)		
d (dense)	17 (13)	18 (13)	5 (25)	4 (22)		
Breast composition					*p* = 0.080	*p* = 0.366
a + b (fatty)	22 (17)	30 (22)	0 (0)	2 (11)		
c + d (dense)	110 (83)	104 (78)	20 (100)	16 (89)		
FGT					*p* = 0.698	*p* = 0.763
a (fatty)	9 (7)	9 (7)	0 (0)	0 (0)		
b	41 (31)	39 (29)	5 (25)	7 (39)		
c	68 (52)	72 (54)	13 (65)	9 (50)		
d (extreme)	14 (11)	14 (10)	2 (10)	2 (11)		
BPE					*p* = 0.558	*p* = 0.394
Minimal	28 (23)	28 (23)	3 (17)	3 (19)		
Mild	37 (30)	39 (32)	8 (44)	6 (38)		
Moderate	34 (28)	35 (28)	3 (17)	2 (13)		
Marked	23 (19)	22 (18)	4 (22)	5 (31)		
PD			OR = 1.02 95% CI = 0.99-1.05	OR = 1.01 95% CI = 0.98-1.04	*p* = 0.277	*p* = 0.553
Breast thickness			OR = 0.98 95% CI = 0.94-1.01	OR = 0.98 95% CI = 0.94-1.02	*p* = 0.205	*p* = 0.237
Lesion characteristics	True positive		False negative			
Size			OR = 0.95 95% CI = 0.90-1.02	OR = 0.93 95% CI = 0.87-1.00	*p* = 0.115	*p* = 0.041
Type of finding					*p* = 0.266	*p* = 1.000
Invasive	64 (77)	63 (74)	13 (65)	14 (78)		
In-situ	19 (23)	22 (26)	7 (35)	4 (22)		
Histological type					*p* = 0.190	*p* = 0.230
Ductal	55 (86)	54 (86)	9 (69)	10 (71)		
Lobular	4 (6)	4 (6)	2 (15)	2 (14)		
Other	5 (8)	5 (8)	2 (15)	2 (14)		
Histological grade					*p* = 0.810	*p* = 0.660
Grade 1	21 (33)	20 (32)	3 (23)	4 (29)		
Grade 2	24 (38)	25 (40)	5 (39)	4 (29)		
Grade 3	19 (30)	18 (29)	5 (39)	6 (43)		
Subtype^b^					*p* = 0.854	*p* = 0.601
Luminal a	36 (56)	36 (57)	9 (69)	9 (64)		
Luminal b HER2-	13 (20)	13 (21)	2 (15)	2 (14)		
Luminal b HER2+	6 (9)	6 (10)	0 (0)	0 (0)		
HER2+ non-luminal	2 (3)	2 (3)	0 (0)	0 (0)		
Triple negative	7 (11)	6 (10)	2 (15)	3 (21)		

*Numbers in parentheses* are percentages

*R* reader, *FGT* fibroglandular tissue, *BPE* background parenchymal enhancement, *PD* percent density, *OR* odds ratio, *CI* confidence interval

^a^Logistic regression and Fisher’s exact test were used for continuous and discrete data, respectively

^b^St Gallen 2013 classification

**Fig. 3:**
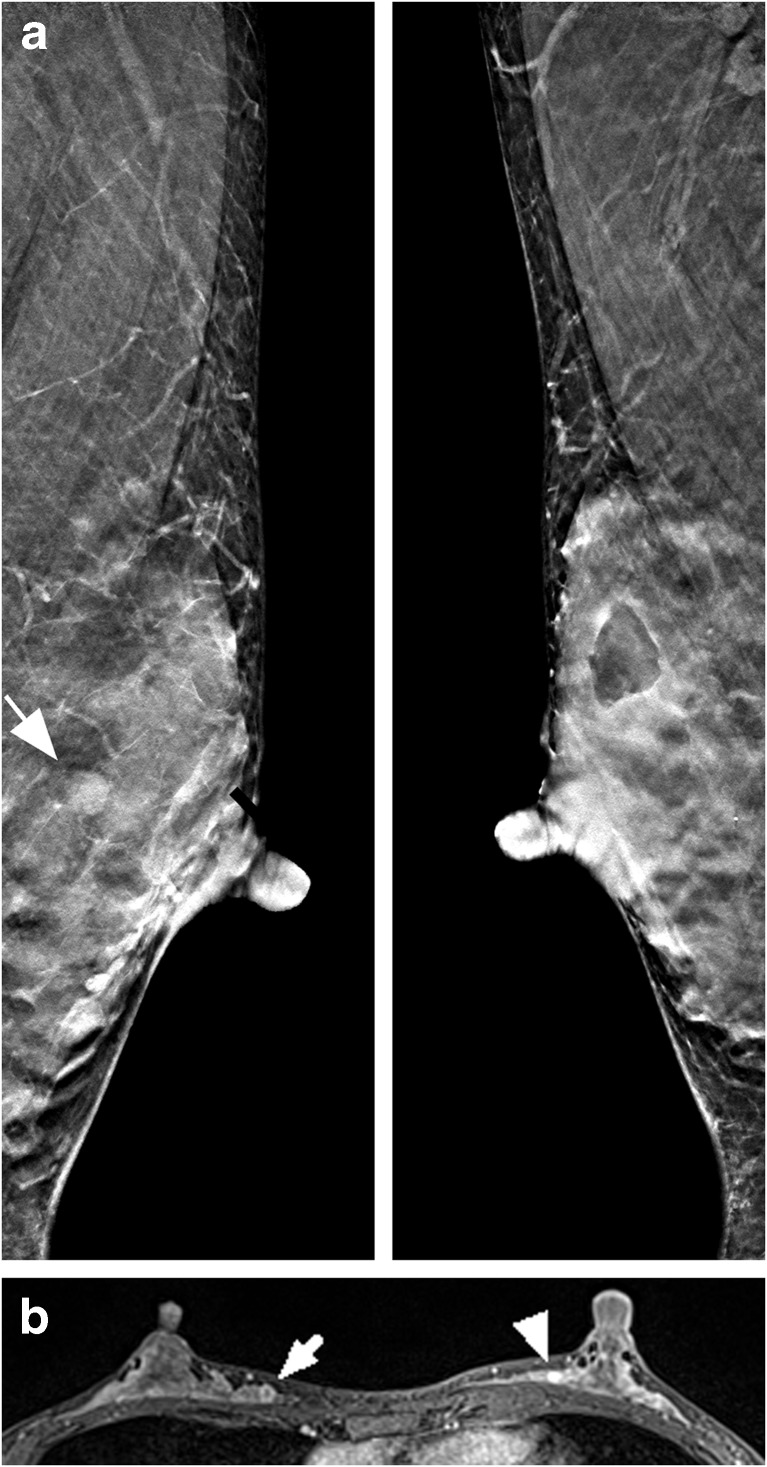
A 37-year-old woman with a 15-mm mucinous carcinoma in the right breast and a benign fibroadenoma in the left breast. **a** Bilateral DBT slices in the mediolateral oblique projection with the fibroadenoma highlighted (*arrow*) in the left breast. Both readers reported breast composition d and percent density was estimated to 70% by Libra. The carcinoma was missed (BI-RADS 1) by both readers and the fibroadenoma was rated BI-RADS 4 by reader 1 and BI-RADS 3 by reader 2. **b** Axial post-contrast T1-weighted MRI with the carcinoma highlighted in the right breast (*arrow*) and the fibroadenoma (*arrowhead*) in the left breast. The amount of fibroglandular tissue was reported heterogeneous and the background parenchymal enhancement was marked. The final report stated BI-RADS 3 finding in the right breast and BI-RADS 4 finding in the left breast

The three FN cases on MRI were as follows: 45-year-old woman with heterogeneous fibroglandular tissue and marked BPE presenting DCIS, BI-RADS 1 (BI-RADS 4 on DBT by both readers), 56-year-old woman with scattered fibroglandular tissue and mild BPE, presenting DCIS, BI-RADS 2 (BI-RADS 4 on DBT by both readers) and 37-year-old woman with heterogeneous fibroglandular tissue and marked BPE presenting a 15-mm, grade 1, luminal a, mucinous carcinoma, BI-RADS 3 (BI-RADS 1 on DBT by both readers) (Fig. 3). Due to the low number of FNs on MRI no statistical tests were performed.

### Lesion characteristics

The mean size 13.2 mm and 11.8 mm of FN was significantly smaller than the mean size 20.6 mm and 21.2 mm of TP on DBT for one reader (R1, *p* = 0.115 and R2, *p* = 0.041). No other cancer characteristics were associated with FN by any reader (*p* > 0.05); however, there was a trend for lobular histological type being missed (Table 2).

**Fig. 4:**
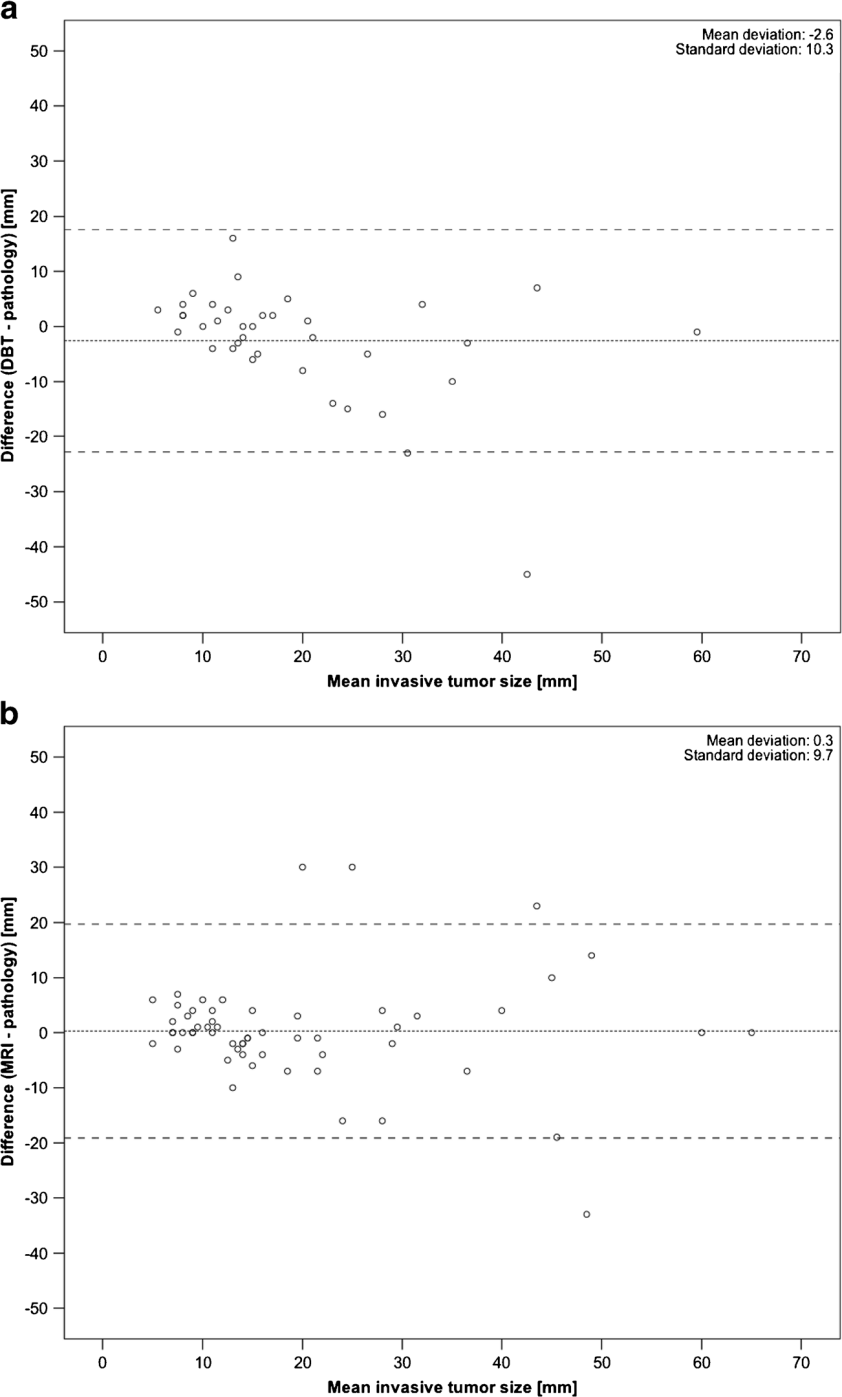
Bland-Altman plot of invasive tumour size measured by pathology subtracted from that measured by DBT (**a**) and MRI (**b**) compared with the mean of the two results. *Middle dashed line* is the mean difference and *top and bottom dashed lines* are the 95% limits of agreement (±2 standard deviations)

Of the 77 invasive masses, 12 underwent neoadjuvant chemotherapy and 1 case metastasised, leaving 64 masses with a pathological mean size of 18.3 mm (median, 15 mm; range, 1-65 mm). DBT could measure the invasive part in 57.8% (37/64) and MRI in 89.1% (57/64) of the cases with size correlation *r* = 0.73 and *r* = 0.78 versus pathology, respectively. One reason for the low DBT percentage was because of the high proportion of masses identified only by the calcification component (*n* = 11) without size comparison to pathology. Figure 4 shows the size deviations of the tumours measured with DBT and MRI compared with the averages of the pathology and said modality, respectively.

## Discussion

The diagnostic performance of MRI was significantly higher compared to DBT for one reader (*p* = 0.004) and of borderline significance for the other reader (*p* = 0.052). Only a few studies have compared the diagnostic performance of DBT versus MRI in specific settings [32–35]. Kim et al. [35] reported higher diagnostic performance (sensitivity, 97.8%) but lower PPV (89.6%) for MRI versus DBT (sensitivity, 88.2%; PPV, 93.3%), which is in line with the results of this study. Clauser et al. [33] and Mariscotti et al. [34] investigated the role of DBT as a so-called second-look modality for additional findings at preoperative MRI, observing an increased detection rate when adding DBT to US, although, still inferior to MRI.

Breast composition, although not statistically significant, tend to be associated with FN on DBT (R1, *p* = 0.080) in contrast to PD. Thus, a masking effect (particularly the use of category c) seems to be prominent regardless of specific percentage density thresholds. The moderate to strong correlation between breast composition and PD also suggests this finding. Since 68% of the women in this study were assigned breast composition c, it would be of great interest to explore any sub categorisation based on, for instance, parenchymal distribution or texture.

The prospective screening trials have not shown a significant cancer detection increase for DBT in category d, owing to the low sample size; however, there was no significant detection increase in breast composition d for DBT compared with DM alone in a multicentre study by Rafferty et al. [36], despite a larger sample size [7–9]. This lack of evidence is the main reason that DBT is still on hold as a complementary tool for the screening of women with dense breasts [22].

Eighty-two percent of the study population had dense breasts as categorised by breast compositions c and d and the density distribution was also significantly different from the anticipated 10%/40%/40%/10% by ACR BI-RADS in a screening setting [17]. As a comparison, data from the Breast Cancer Surveillance Consortium [37] showed that 43.3% of the US women are considered dense. The distribution of PD, as estimated by Libra within the radiologist-provided breast composition categories (Fig. 1.), resembled those in the work of Keller et al. [27], except for category c demonstrating a broader range. This is likely a result of the adoption of the new ACR BI-RADS category c definition, taking into account the masking effect. Comparing this Japanese study population (mean age, 57 years) with black and white Pennsylvanian women (*n* = 9,498; mean age, 57 years) regarding PD estimations using Libra, the population in this study had more than double PD, 44% versus 12.3% (black) and 17.1% (white) [38]. Despite not being a screening population, this result supports the view that Japanese women have dense breasts [12].

The literature is sparse regarding correlation of qualitative imaging assessments of FGT, BPE and mammographic breast composition. Hansen et al. [39] found a correlation of 0.36 between BPE and mammographic breast composition compared to 0.28 in this study. King et al. [40] found a non-significant correlation for one reader and a significant correlation of 0.4 for another between FGT and BPE, compared to 0.37 in this study. No studies were found correlating qualitative FGT and mammographic breast composition, which were considered moderate in this study (*r* = 0.53 and *r* = 0.54 for R1 and R2, respectively).

The preoperative size assessment was superior for MRI compared to DBT, showing a stronger correlation and a better size agreement with pathology (Fig. 4.). The DBT correlation coefficient of 0.73 was a little lower compared with other published studies: 0.86 by Förnvik et al. [29] and 0.86 by Luparia et al. [30], which could be explained by the predominantly dense breast population or the use of a narrow-angle DBT system. Chudgar et al. [41] assessed the use of preoperative MRI for disease extent in breast cancer detected at DBT versus DM alone and found that women with fatty breasts screened with DBT may benefit less from preoperative MRI than women with more dense breasts. By applying a PD threshold based on the FN, about 10% (13/137) of the study population could potentially undergo DBT instead of MRI without any loss in sensitivity. Using breast composition groups a and b would increase specificity at a slight cost of sensitivity.

A limitation of the current study was the exclusion of BI-RADS 1 and 2 cases because of the sporadic follow-up in these groups, especially in the tertiary setting. Consequently, this could bias PPV in favour of DBT and overestimate MRI sensitivity. The relatively short minimum follow-up of 1 year for negative cases not undergoing biopsy/surgery is also a limitation of the current study. This study has followed the recommendations of BI-RADS 5th edition; thus, care should be taken when comparing the results with older BI-RADS editions, particularly regarding the breast density classification. The study population may also differ between a tertiary setting and a non-tertiary setting with regard to breast cancer profile and breast density. Another limitation was the final decision-making by one breast specialist for the MRI interpretation limiting the generalisation of the results. In addition, priors were not read during DBT interpretation, which could lower the diagnostic performance. It was not within the scope of this paper to compare the added value of DBT to DM alone.

In conclusion, DBT can be performed instead of MRI in women with non-dense breasts as determined by either a PD threshold or breast compositions a and b or in the case of MRI contraindications. MRI has higher diagnostic performance than DBT in a dense breast population in the tertiary setting.

## Abbreviations

DBT: Digital breast tomosynthesis
PD: Percent density
DM: Digital mammography
FGT: Fibroglandular tissue
BPE: Background parenchymal enhancement
FN: False negative
NST: No special type
DCIS: Ductal carcinoma in situ
ILC: Invasive lobular carcinoma
FP: False positive
